# Comparative functional, perioperative and oncological outcomes of robot-assisted and open radical prostatectomy: a systematic review and meta-analysis

**DOI:** 10.1007/s11255-026-05182-5

**Published:** 2026-06-02

**Authors:** Caio Vinicius Suartz, Mateus Silva Santos Costa, Caio Felipe Araujo Matalani, Marcelo Ribeiro da Rocha, José Pedro Cassemiro Micheleto, Pedro Luis Furlam, Henrique Laurent Lepine, Roberto Iglesias Lopes, Thalita Bento Talizin, José Bessa Júnior, Walid Shahrour, Walid Shabana, Axel S. Merseburger, Leonardo Oliveira Reis, Maurício Dener Cordeiro, William Carlos Nahas, Paul Toren, Leopoldo Alves Ribeiro-Filho, Guillaume Ploussard, Vincent Fradet, Frédéric Pouliot

**Affiliations:** 1 Urology Department, Northern Ontario School of Medicine, 955, Oliver Road, Thunder Bay, ON Canada; 2 CHU de Québec-Université Laval, Quebec City, QC Canada; 3 Division of Urology, Institute of Cancer of São Paulo, University of São Paulo, São Paulo, Brazil; 4 Research Department, Brazilian Society of Urology, São Paulo, Brazil; 5 Department of Health, State University of Feira de Santana (UEFS), Feira de Santana, BA Brazil; 6 Department of Urology, Universitätsklinikum Schleswig–Holstein, Lübeck, Germany; 7 UroScience, School of Medical Sciences, State University of Campinas, Unicamp, and ImmunOncology, Pontifical Catholic University of Campinas, PUC-Campinas, Campinas, SP 13083-872 Brazil; 8 Department of Urology, La Croix du Sud Hospital, Quint Fonsegrives, France

**Keywords:** Prostate cancer, Robot-assisted radical prostatectomy, Open radical prostatectomy, Randomized controlled trial, Meta-analysis, Functional outcomes, Oncological outcomes, Perioperative outcomes

## Abstract

**Introduction:**

Robot-assisted radical prostatectomy (RARP) has increasingly replaced open radical prostatectomy (ORP) based on evidence suggesting improved perioperative outcomes and faster recovery. However, few randomized trials directly compare both techniques. This review compares perioperative, functional, and oncological outcomes of RARP versus ORP, integrating newly available high-impact studies.

**Methods:**

A thorough search of major databases was conducted from the inception of each database up to November 2025, adhering to PRISMA guidelines. Two reviewers independently screened studies in a three-step process—titles, abstracts, and full texts. Only randomized controlled trials (RCTs) and prospective studies comparing RARP and ORP were included. All variables were entered into a spreadsheet for analysis and cross-checked. The primary endpoints assessed were perioperative complications, functional recovery (urinary continence and erectile function), and oncological outcomes.

**Results:**

A total of 27 studies comprising 38,530 patients were included. Compared with ORP, RARP was associated with significantly reduced intraoperative blood loss (mean difference [MD] :  − 576.78; *p* < 0.01), lower transfusion rates (Odds Ratio [OR] 0.27; *p* < 0.0001), and shorter hospital stay (MD:   − 1.44 days; *p* < 0.0001). RARP also demonstrated fewer postoperative complications (OR 0.59; *p* = 0.002). Regarding functional outcomes, RARP yielded superior urinary continence recovery at 3 months and improved erectile function recovery (OR 1.43). Additionally, RARP showed a lower biochemical recurrence rate at 24 months (OR 1.34), with no significant difference observed in short-term follow-up (< 12 months).

**Conclusion:**

RARP offers advantages over ORP, particularly in reducing perioperative morbidity and enhancing functional recovery while maintaining comparable or superior oncological outcomes. However, further randomized controlled trials with extended follow-up are necessary to confirm these findings.

**Key messages:**

1. RARP was associated with significantly reduced blood loss, lower transfusion requirements, and shorter hospitalization.
2. Functional outcomes favored RARP, including superior recovery of urinary continence and higher rates of nerve-sparing preservation.
3. Overall, the evidence supports RARP as a safe, effective, and clinically advantageous approach for localized prostate cancer.

**Supplementary Information:**

The online version contains supplementary material available at https://doi.org/10.1007/s11255-026-05182-5.

## Introduction

The surgical treatment of prostate cancer has seen significant advancements in recent decades. Since Binder et al. (2001), the robot-assisted radical prostatectomy (RARP) has revolutionized surgical management, being the primary surgical approach in many countries [1–6], gaining space over open radical prostatectomy (ORP) and laparoscopic prostatectomy. This new approach is believed to have improved functional outcomes, reduced perioperative complications, decreased blood loss and transfusion rates, and shortened length of hospital stays [7, 8].

Despite these advances, few randomized controlled trials (RCTs) have been published in prostate cancer comparing ORP with RARP. This type of trial is imperative in providing high-quality evidence about the efficacy of the different treatment modalities. Recently, Nahas et al. (2024) [9] published an RCT, which furnished valuable new data that could play a pivotal role in informing clinical recommendations and healthcare policies. Specifically, the trial demonstrated that RARP leads to faster urinary continence recovery, with a significant advantage observed at the 3- and 6-month postoperative assessments. Furthermore, contrary to recent evidence [10], the study reported no statistically significant differences in blood transfusion rates, overall complications, or erectile function recovery.

Although relevant reviews with comparable study selection criteria have already been published, such as the one conducted by Wang et al. (2023) [10], the publication of recent high-impact studies, including that by Nahas et al. (2024) [9], prompted the present systematic review. This review specifically includes prospective cohort studies and RCTs published up to November 2025 that meet the inclusion criteria involving patients undergoing RARP or ORP, with the objective of comparing the perioperative, functional, and oncological outcomes of RARP versus ORP, integrating newly published high-impact studies on the topic and providing a more robust foundation for clinical decision-making.

## Materials and methods

### Literature search

A comprehensive search was performed up to November 2025 in the three major databases—Cochrane Central Register of Controlled Trials, MEDLINE, and Embase. The research was completed in full compliance with the Preferred Reporting Items for Systematic Reviews and Meta-Analysis (PRISMA) [11] statement on December 2025. It was registered in the PROSPERO international database as a prospectively registered systematic review (CRD 42024568284).

A research question was formulated based on the Patient-Intervention-Comparator-Outcome-Study (PICOS) criteria: [12] Among patients undergoing radical prostatectomy for localized prostate cancer, which surgical approach—RARP or ORP—provides superior functional, oncological, and perioperative outcomes?

The search strategy can be found in the supplementary material (Supplementary File 1).

### Study screening and selection

Two reviewers (MC—urology resident and CM—general surgery resident) independently screened studies in a three-step process—titles, abstracts, and full texts. Reference lists of the included articles were searched to identify additional studies through citation tracking. Discrepancies were resolved through discussion with a third investigator (CS—urologist). All screening and data-extraction activities were managed using the Rayyan platform [13]. Data were extracted and plotted into a spreadsheet using Microsoft Excel 2019 (Microsoft Corporation, Redmond, WA).

### PICOS framework

We included studies that met the following criteria: (1) patients (P)—adults over 18 years old with clinically localized prostate cancer; (2) the intervention (I) analyzed was RARP, and the comparator (C) was ORP. The primary outcomes (O) evaluated were functional and oncological results, and the search was done including only prospective studies and randomized controlled trials (S).

### Inclusion and exclusion criteria

Prospective observational studies and RCTs were included. Meeting abstracts, reviews, case reports, letters to the editor, retrospective cohorts, and editorials were excluded.

### Data extraction and endpoints

All the variables were integrated into a spreadsheet for analysis and cross-checked by another author (Table 1). The mean and standard deviation for continuous variables were recorded from the included studies. For variables reported as median and interquartile range, the original data were converted to mean and standard deviation [14].

**Table 1:** Variables included in each study

Study	Variables included
Nahas, W.C (2024) [9]	2, 3, 4, 5, 6, 7, 8, 9, 10, 11, 12, 13
Wallerstedt Lantz, A (2019) [38]	1, 2, 3, 7, 8
Sooriakumaran, P (2018) [39]	1, 2, 3, 10, 11, 12, 13
Di Pierro, G. B (2011) [21]	2, 3, 4, 8, 9, 10, 11, 12, 13
Doumerc, N (2010) [29]	1, 2, 3, 4, 5, 7, 8, 9, 11
Barocas, D.A (2010) [22]	2, 3, 11, 12
Cortes, A. G (2009) [32]	1, 2, 3, 4, 5, 6, 7, 8, 9, 10, 11, 12, 13
Lantz, A (2021) [40]	1, 2, 3, 9, 10, 11, 12, 13
Chang, P (2022) [36]	1, 2, 3, 5, 6, 7, 8, 13
Raheem, A.A (2020) [23]	1, 2, 3, 5, 6, 7, 8, 9, 11, 12, 13
Capogrosso, P (2017) [25]	2, 3, 10, 13
Coughlin, G.D (2018) [20]	9, 10, 12
Yaxley, J.W (2016) [19]	2, 3, 4, 5, 6, 7, 8, 9, 10, 11
Nyberg, M (2018) [43]	1, 2, 3, 9, 10, 12, 13
Capogrosso, P (2016) [24]	9, 10, 13
Ong, W.L (2015) [26]	2, 3, 9, 10, 11, 12
Haglind, E (2015) [33]	2, 3, 4, 5, 6, 9, 10, 11, 13
Davison, B.J (2014) [30]	2, 3, 9, 10
Geraerts, I (2013) [27]	2, 3, 9, 11, 13
Ludovico, G. M (2013) [41]	2, 3, 4, 5, 6, 8, 9, 10, 11, 13
Hong, J.Y (2010) [28]	3, 4, 6, 7
Rocco, B (2009) [31]	2, 3, 4, 5, 6, 9, 10, 11, 13
Ficarra, V (2009) [8]	1, 2, 3, 4, 5, 6, 7, 8, 9, 10, 11, 13
Ham, W.S (2008) [37]	2, 3, 5, 6, 8, 9, 10, 11, 13
Choo, M.S (2013) [34]	1, 2, 3, 4, 6, 7, 9, 10, 11, 12, 13
Hagman, A (2021) [42]	1, 2, 3, 9, 11, 12, 13
Haese, A (2019) [35]	2, 3, 4, 5, 6, 7, 9, 10, 11, 12, 13

1: prostate volume; 2: Gleason; 3: staging; 4: operative time; 5: hospital stay; 6: blood loss; 7: transfusion rate; 8: complications; 9: urinary continence; 10: erectile dysfunction; 11: positive surgical margin rate; 12: biochemical recurrence; 13: nerve sparing

The recorded variables included the median follow-up of the studies, number of patients, body mass index, age, preoperative PSA and prostate volume, clinical Gleason score and TNM stage, pathological Gleason score and TNM stage, surgery duration, length of hospital stay, perioperative blood loss and transfusion rate, postoperative complications stratified by the Clavien–Dindo classification [15], post-operative urinary continence and erectile function, catheterization time and nerve-sparing rate.

Minor complications were defined as Clavien–Dindo grades I and II, while major complications were defined as Clavien–Dindo grades III to V. Functional and specific perioperative complications were recorded as dichotomous variables, including rectal damage, anastomotic leakage, lymphocele, lymphedema, stricture, bladder neck contracture, urinary retention, intensive care unit requirement, deep vein thrombosis, ileus and pulmonary embolism. Intraoperative and oncological variables were recorded as continuous variables, including operative time, number of nodes retrieved, number of positive lymph nodes, period with drain, recurrence after surgery, and overall survival.

### Quality assessment and risk of bias

The risk of bias was analyzed in the prospective studies using ROBINS-1 [16]; and RCTs were appraised with ROB-2 [17], both are tools developed by the Cochrane Collaboration for assessing the risk of bias in non-randomized studies of interventions and randomized trials, respectively. The GRADE system was used to assess the evidence for the outcomes analyzed in the systematic review.

### Statistical analysis

This systematic review with meta-analysis was performed in accordance with the Cochrane Collaboration and the Preferred Reporting Items for Systematic Reviews and Meta-Analysis (PRISMA) statement guidelines [18] utilizing the Review Manager 5.4 (Cochrane Centre, The Cochrane Collaboration, Denmark). Odds ratios (OR) with 95% confidence intervals were used to compare treatment effects for categorical endpoints. Cochran Q test and I^2^ statistics were used to assess for heterogeneity; *p* values inferior to 0.10, and I^2^ > 25% were considered significant for heterogeneity. We used a fixed-effect model for outcomes with low heterogeneity (I^2^ < 25%). Otherwise, a DerSimonian and Laird random-effects model was used.

Sensitivity analyses were conducted to evaluate the robustness and reliability of the pooled effect estimates. A leave-one-out approach was applied, whereby each study was sequentially removed to assess its influence on the overall effect size and between-study heterogeneity. Additional sensitivity analyses were performed by restricting the dataset to studies with low risk of bias, as well as according to predefined methodological criteria, including study design and outcome definitions. Stability of the results across these analyses was considered indicative of the consistency and methodological soundness of the meta-analytic findings.

## Results

### Literature screening

**Fig. 1:**
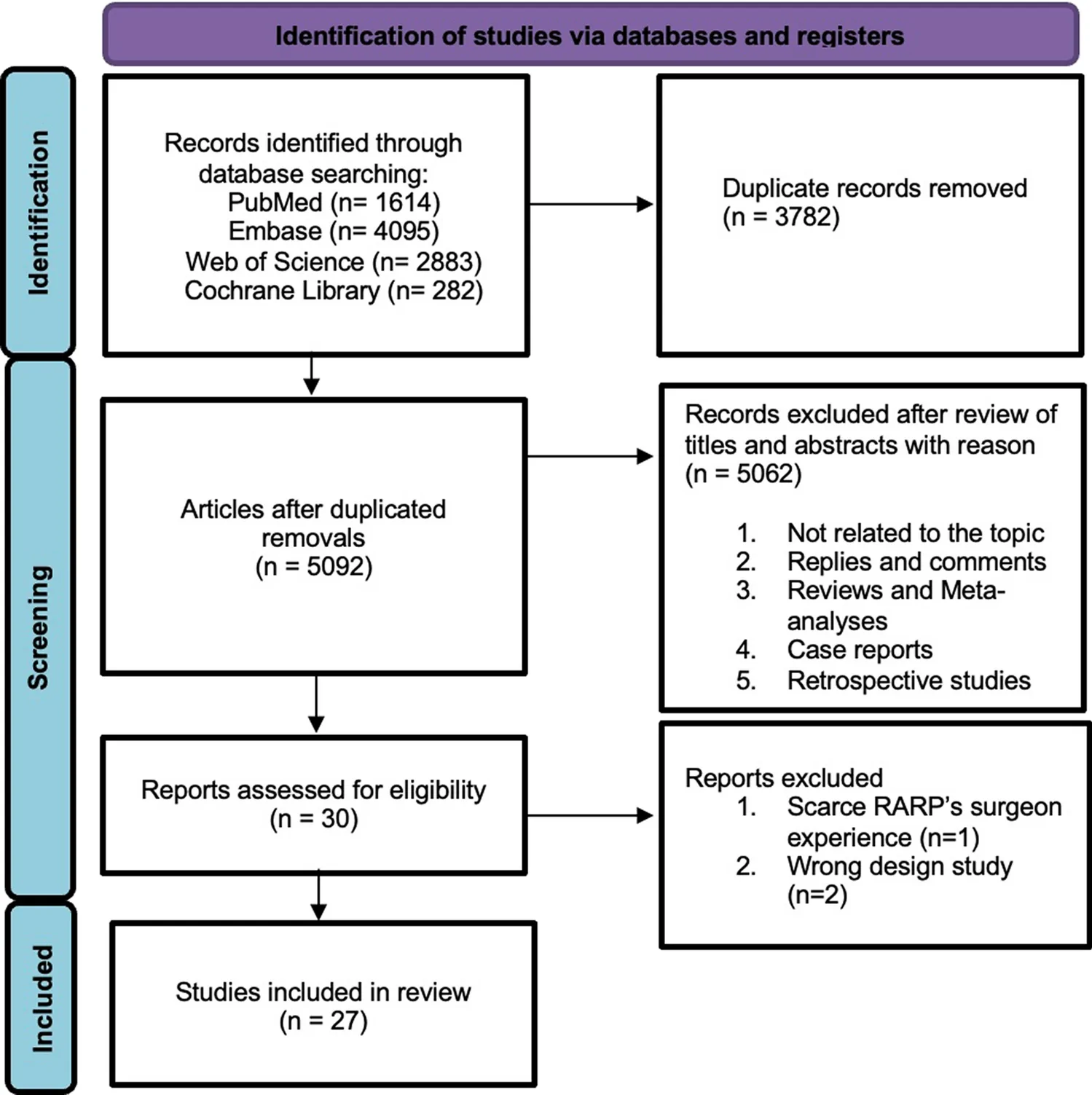
PRISMA flowchart of the literature search

The literature search retrieved 8874 studies. After the automatic exclusion of duplicates, 5092 studies remained. Following the title and abstract reading, 5062 articles were excluded for incompatibility with the study’s aim. Thirty articles were selected for full-text reading. Twenty-seven were selected and included for statistical analysis. One discarded study was not added to the review because it did not require prior experience from the RARP group surgeon, which increased the risk of confounding bias, once all other studies only included surgeries performed by experienced surgeons. Two other studies were retrospective but did not make this clear in the abstract, making it possible to exclude them only after full-text reading. Figure 1 shows the PRISMA flowchart of the literature search.

### Quality assessment

**Fig. 2:**
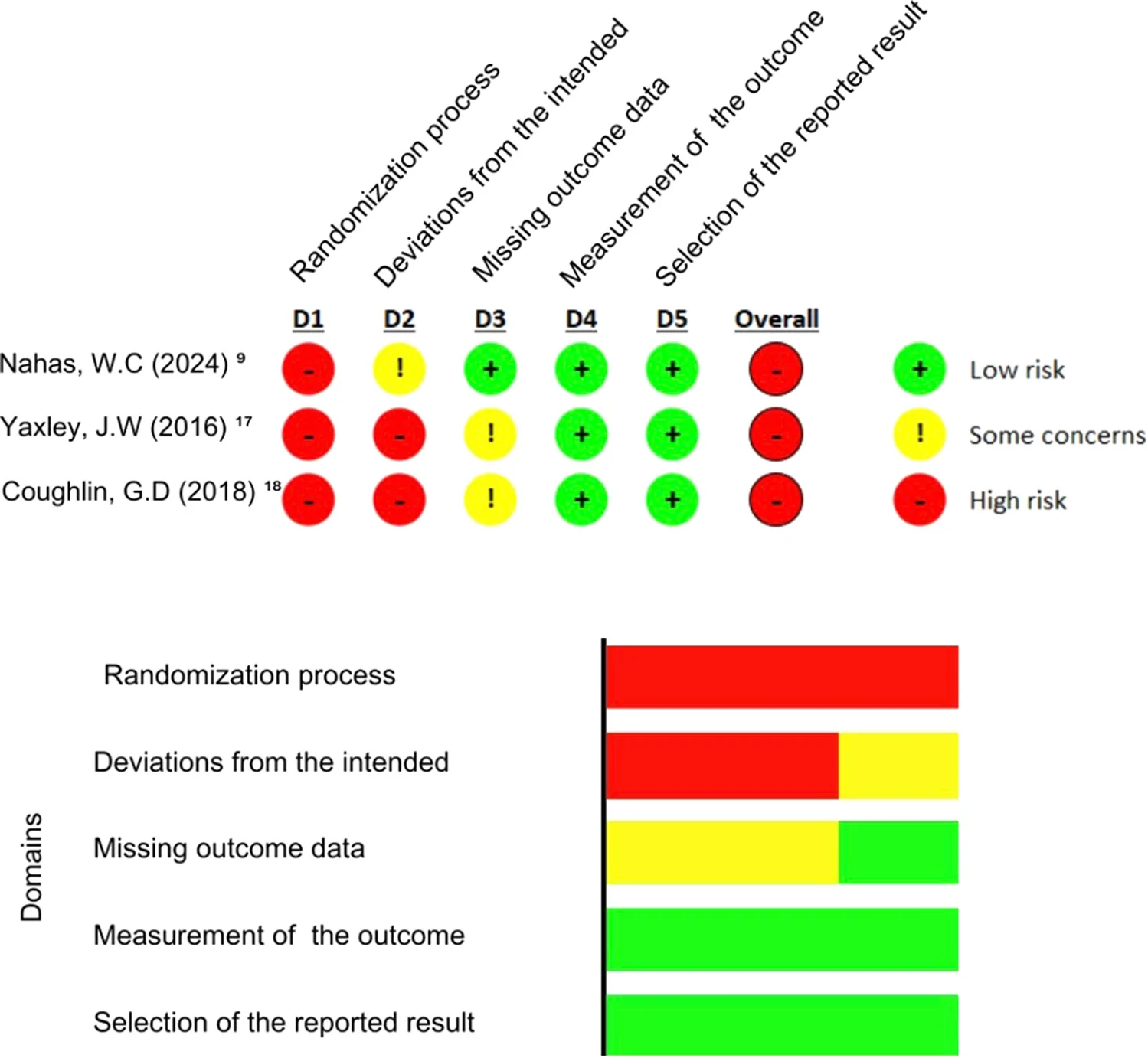
Risk of Bias 2 (RoB 2) tool

The GRADE system was used to assess the evidence for the outcomes analyzed in the systematic review (Fig. 2). GRADE Pro-GDT exported the summary table with the certainty of evidence ratings. The studies were classified as observational cohorts due to the high proportion of prospective non-randomized studies in the analysis and the limited number of included RCTs (Supplementary material).

The selected studies had different designs, with only two studies being RCTs.

The RCT studies were classified as high risk in D1 due to the lack of blinding. Yaxley et al. [19]/ Coughlin et al. [20] were deemed high risk in D2 due to significant patient losses during follow-up and patient dropouts after allocation. In Nahas et al. [9], despite patient losses during follow-up, 15 patients allocated to the ORP group did not undergo surgery, resulting in a ‘Some Concerns’ classification in D2.

None of the studies employed double-blinding, but in the studies by Yaxley et al. [19] and Coughlin et al. [20] the pathologists and researchers involved in data analysis were unaware of the surgical approach used for each patient. In Nahas et al. [9], an attempt to reduce observer bias was made by having nurses not affiliated with the urology department collect the data. Thus, these two studies represent the highest quality assessment among the evaluated studies.

### Risk of bias

Regarding the ROBINS-I evaluation, most studies exhibited a moderate level of bias overall, predominantly due to confounding, measurement of outcomes, and the selection of reported results. These biases are often inherent in non-randomized studies comparing robotic versus open radical prostatectomy, where uncontrolled confounding factors and inconsistencies in outcome measurement are common. The moderate bias observed, particularly in confounding, suggests that patient differences, such as baseline health status and disease severity, may not have been adequately accounted for. Similarly, bias in the measurement of outcomes, such as functional recovery and oncological success, and bias in the selective reporting of results may influence the comparability of findings across studies. These limitations emphasize the need for cautious interpretation of results and highlight the importance of robust randomized controlled trials to mitigate these biases and validate the reported outcomes, as shown in Table 2.

**Table 2:** Risk of bias in non-randomized studies—ROBINS-I tool

Study	Confounding	Selection bias	Bias in classification of interventions	Deviations from intended interventions	Bias due to missing data	Bias in measurement of outcomes	Bias in selection of reported result	Overall
Wallerstedt Lantz, A (2019) [38]	Low	Low	Low	Low	Low	Moderate	Low	Low
Sooriakumaran, P (2018) [39]	Low	Low	Low	Low	Moderate	Moderate	Moderate	Moderate
Di Pierro, G. B (2011) [21]	Moderate	Low	Low	Moderate	Moderate	Moderate	Low	Moderate
Doumerc, N (2010) [29]	Moderate	Low	Low	Low	Low	Low	Moderate	Moderate
Barocas, D.A (2010) [22]	Moderate	Low	Low	Low	Low	Low	Moderate	Moderate
Cortes, A. G (2009) [32]	Moderate	Low	Low	Moderate	Moderate	Moderate	Low	Moderate
Lantz, A (2021) [40]	Low	Low	Low	Low	Low	Low	Low	Low
Chang, P (2022) [36]	Moderate	Low	Low	Low	Low	Low	Moderate	Moderate
Raheem, A.A (2020) [23]	Moderate	Low	Low	Moderate	Moderate	Moderate	Low	Moderate
Capogrosso, P (2017) [25]	Low	Low	Moderate	Moderate	High	Moderate	Low	Moderate
Nyberg, M (2018) [43]	Low	Low	Low	Low	Moderate	Low	Low	Low
Capogrosso, P (2016) [24]	Low	Low	Moderate	Moderate	Moderate	Moderate	Low	Moderate
Ong, W.L (2015) [26]	Moderate	Low	Low	Low	Low	Moderate	Low	Low
Haglind, E (2015) [33]	Low	Low	Low	Low	Moderate	Low	Low	Low
Davison, B.J (2014) [30]	Moderate	Low	Moderate	Low	Low	Moderate	Moderate	Moderate
Geraerts, I (2013) [27]	Moderate	Low	Low	Low	Low	Moderate	Low	Low
Ludovico, G. M (2013) [41]	Moderate	Low	Low	Low	Moderate	Moderate	Moderate	Moderate
Hong, J.Y (2010) [28]	Moderate	Low	Low	Low	Low	Low	Low	Low
Rocco, B (2009) [31]	Moderate	Low	Low	Low	Low	Low	Moderate	Moderate
Ficarra, V (2009) [9]	Moderate	Low	Low	Low	Moderate	Low	Low	Moderate
Ham, W.S (2008) [37]	Moderate	Low	Low	Low	Moderate	Moderate	Low	Moderate
Choo, M.S (2013) [34]	Moderate	Low	Low	Low	Low	Moderate	Moderate	Moderate
Hagman, A (2021) [42]	Moderate	Low	Low	Low	Low	Moderate	Low	Moderate
Haese, A (2019) [35]	Moderate	Low	Low	Low	Moderate	Low	Moderate	Moderate

### Pooled analysis of all studies

All studies compared patients undergoing RARP with ORP. A total of 38,530 patients were included, with 18,199 undergoing ORP and 20,331 undergoing RARP. Table 3 presents the baseline characteristics of the included studies.

**Table 3:** Baseline characteristics of the included studies

Study	Country	Study period	Study design	Number of patients (ORP/RARP)
Nahas, W.C (2024) [9]	Brazil	2014–2021	RCT	156/171
Wallerstedt Lantz, A (2019) [38]	Sweden	2008–2011	Prospective Cohort	2764/942
Sooriakumaran, P (2018) [39]	Sweden	2008–2011	Prospective Cohort	753/1792
Di Pierro, G. B (2011) [21]	Switzerland	2007–2009	Prospective Cohort	75/75
Doumerc, N (2010) [29]	Australia	2006–2008	Prospective Cohort	212/502
Barocas, D.A (2010) [22]	USA	2003–2008	Prospective Cohort	491/1413
Cortes, A. G (2009) [32]	Spain	2011–2018	Prospective Cohort	122/390
Lantz, A (2021) [40]	Sweden	2008–2011	Prospective Cohort	885/2699
Chang, P (2022) [36]	USA	2003–2006	Prospective Cohort	545/549
Raheem, A.A (2020) [23]	Egypt	2013–2016	Prospective Cohort	59/125
Capogrosso, P (2017) [25]	Italy	2003–2013	Prospective Cohort	67/67
Nyberg, M (2018) [43]	Sweden	2008–2011	Prospective Cohort	778/1847
Capogrosso, P (2016) [25]	Italy	2003–2013	Prospective Cohort	395/354
Coughlin, G.D (2018) [20]	Australia	2010–2014	RCT	146/150
Yaxley, J.W (2016) [19]	Australia	2010–2014	RCT	151/157
Ong, W.L (2015) [26]	Australia	2009–2012	Prospective Cohort	1117/885
Haglind, E (2015) [33]	Sweden	2008–2011	Prospective Cohort	778/1847
Davison, B.J (2014) [30]	Canada	2007–2009	Prospective Cohort	73/78
Geraerts, I (2013) [27]	Belgium	2009—2011	Prospective Cohort	64/180
Ludovico, G. M (2013) [41]	Italy	2004–2008	Prospective Cohort	48/82
Hong, J.Y (2010) [28]	South Korea	2007–2007	Prospective Cohort	25/26
Rocco, B (2009) [31]	Italy	2004–2007	Prospective Cohort	240/120
Ficarra, V (2009) [8]	Italy	2006–2007	Prospective Cohort	105/103
Ham, W.S (2008) [37]	South Korea	2002–2007	Prospective Cohort	110/188
Choo, M.S (2013) [34]	South Korea	2003–2010	Prospective Cohort	176/77
Hagman, A (2021) [42]	Sweden	2008–2011	Prospective Cohort	805/1845
Haese, A (2019) [35]	Germany	2008–2016	Prospective Cohort	7007/3783

*RCT* randomized control trial, *ORP* open radical prostatectomy, *RARP* robot-assisted radical prostatectomy

### Tumor staging

Tumor staging varied across the studies, with cT2 tumors being the most prevalent. Ten studies did not report clinical staging [19–28]. Three studies included patients with clinically positive nodal status, totaling 257 cases in the ORP group and 176 cases in the RARP group [9, 21, 29].

Regarding the Gleason score and ISUP classification from prostate biopsy, there was also heterogeneity in reporting, making it difficult to accurately distinguish cases between subgroups with a score of seven or less. Five articles did not mention the patients’ Gleason scores [21, 24, 27, 28, 30]. From the available results, most surgeries were performed on patients with a Gleason score of seven or less in both groups, as shown in Table 4.

**Table 4:** Clinical and pathological characteristics of the ORP and RARP groups

Variable	ORP	%	RARP	%	Total
Gleason (biopsy)					
≤ 7	8154	91.09%	16,337	93.18%	24,491
> 8	798	8.91%	1195	6.82%	
cT stage					
cT1	4269	62.77%	8546	58.95%	13,451
cT2	2305	33.89%	5534	38.17%	8076
≥ cT3	227	3.34%	417	2.88%	653
Clinical nodal status (cN)					
cN +	257	1.48%	176	0.86%	433

*ORP* open radical prostatectomy, *RARP* robot-assisted radical prostatectomy

### Peri-operative outcomes

#### Surgical duration

**Fig. 3:**
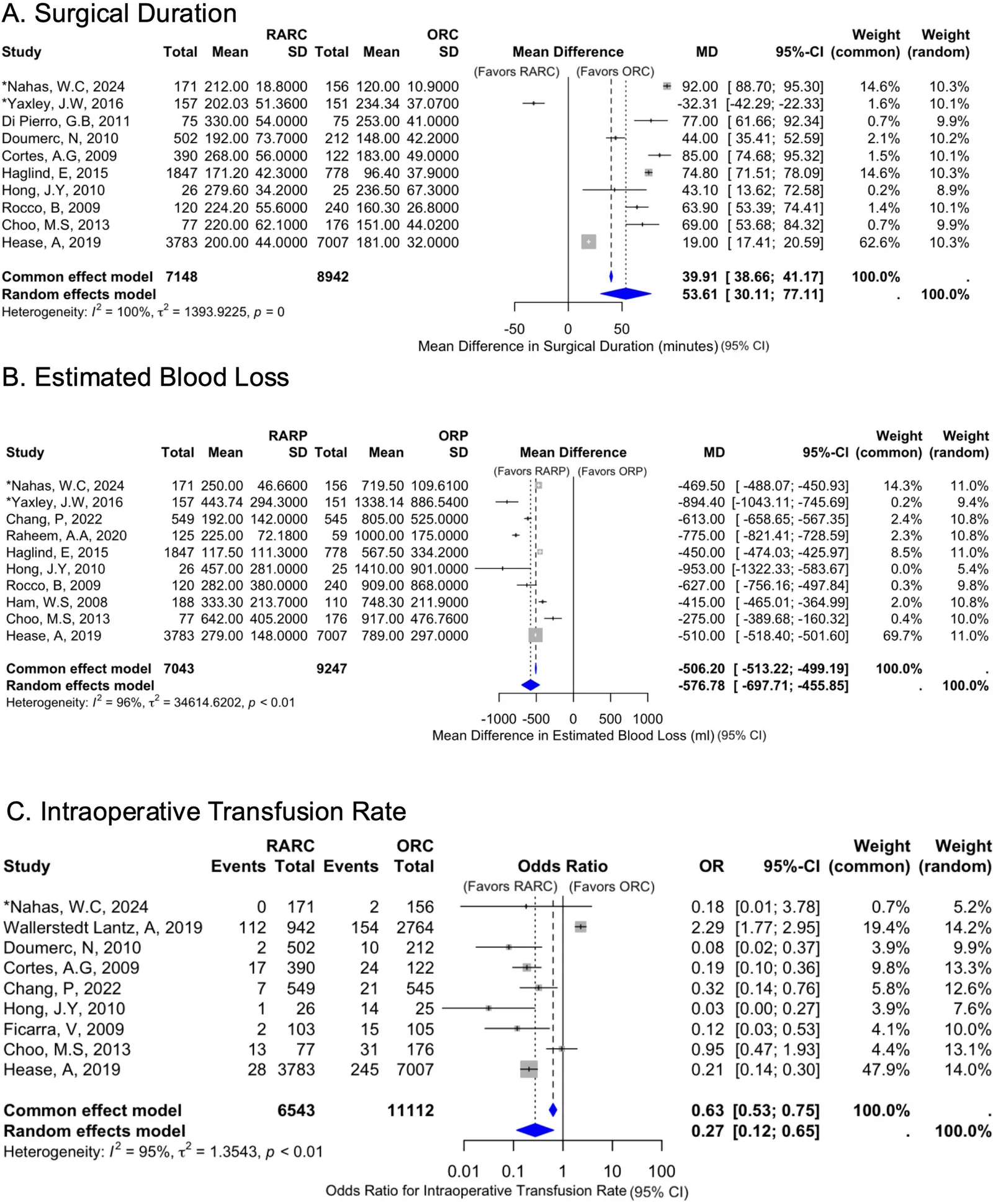

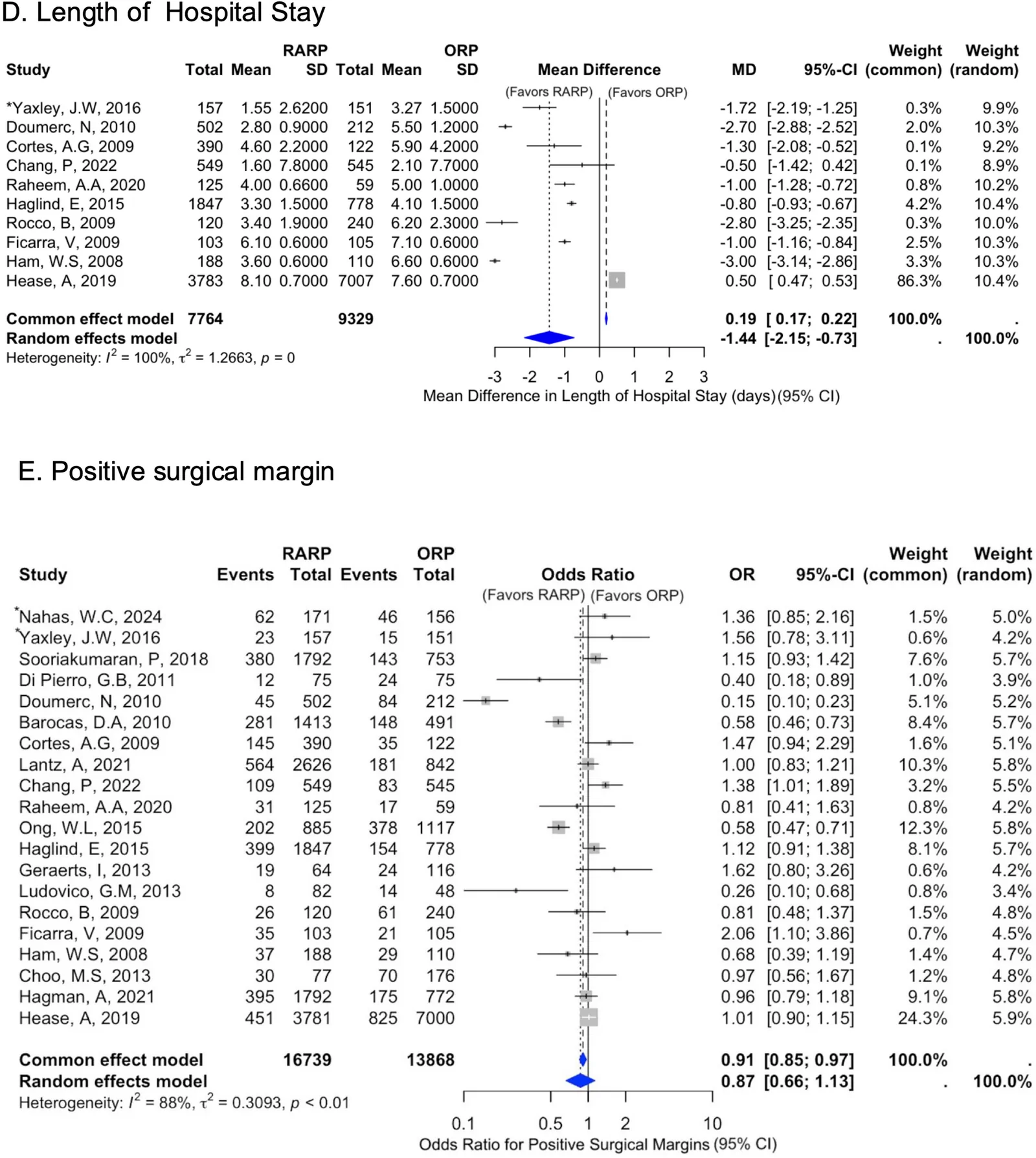

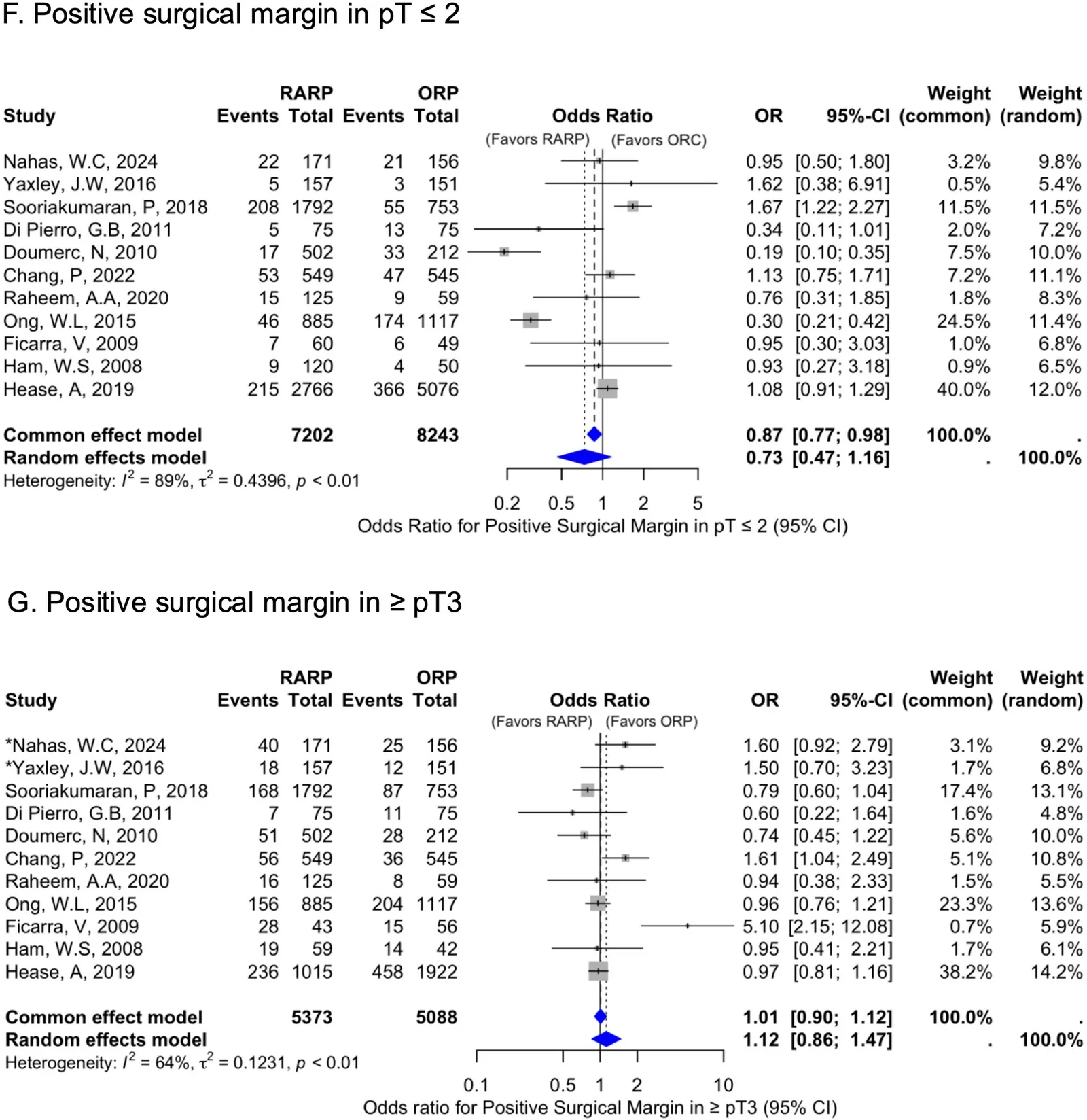

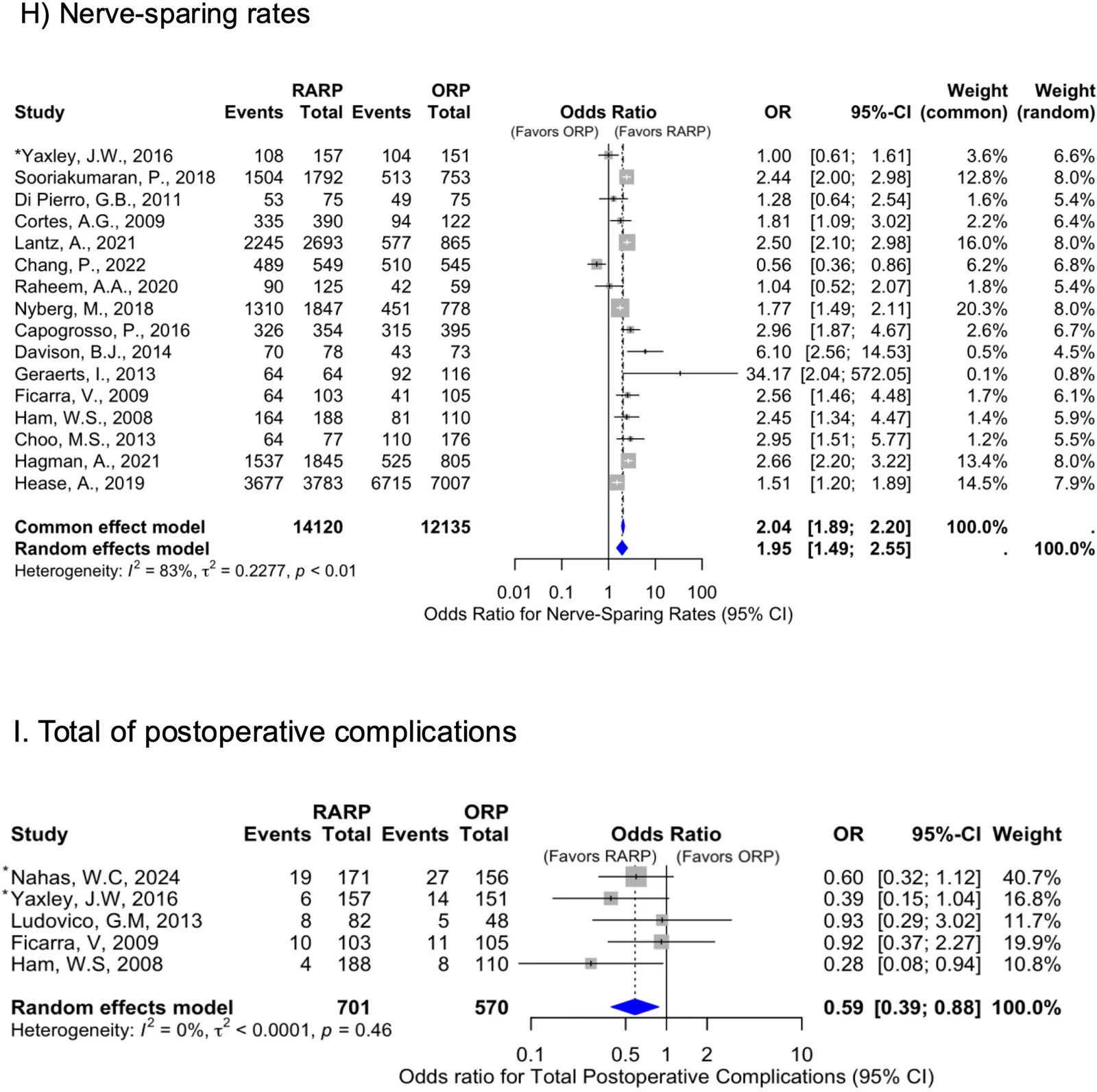

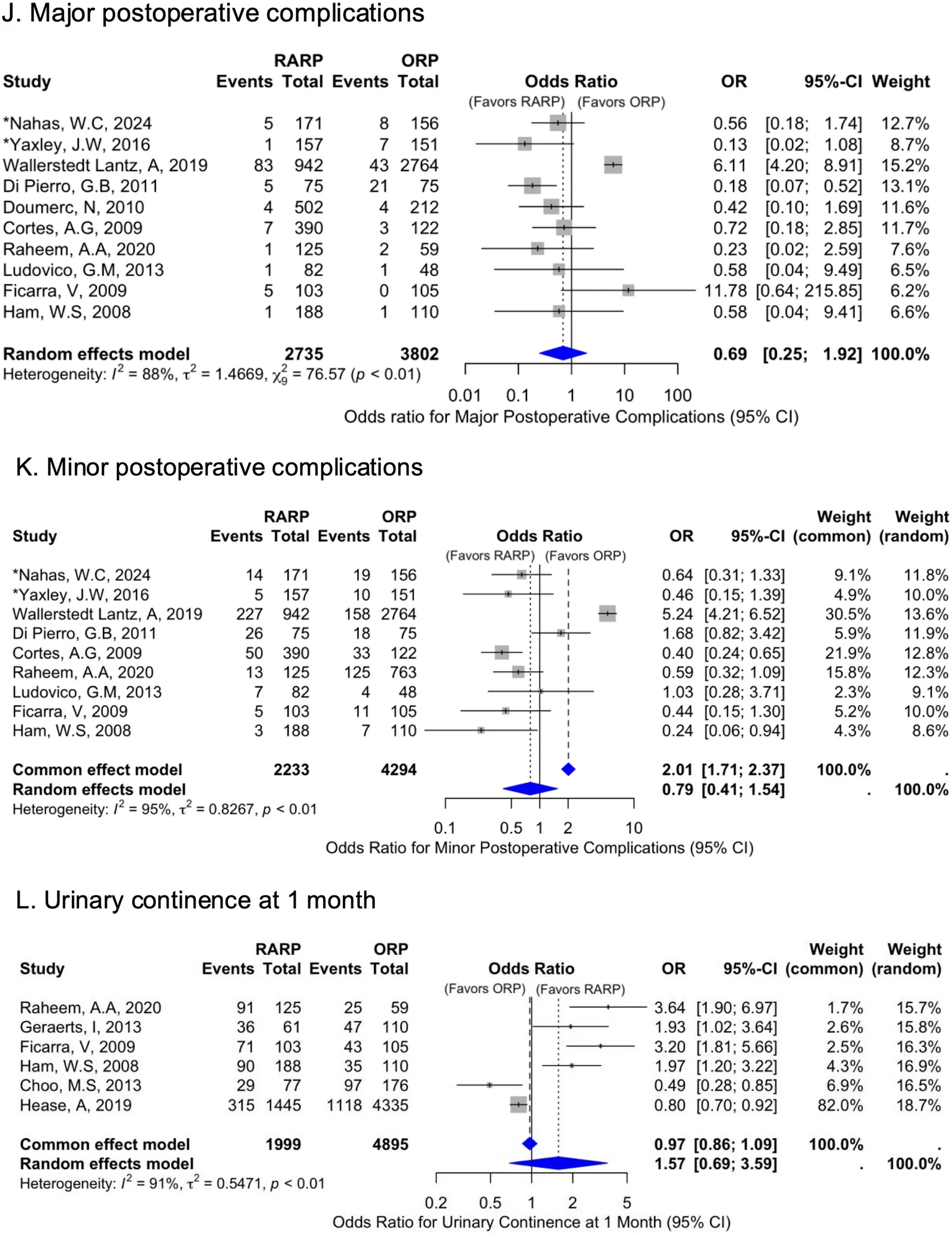

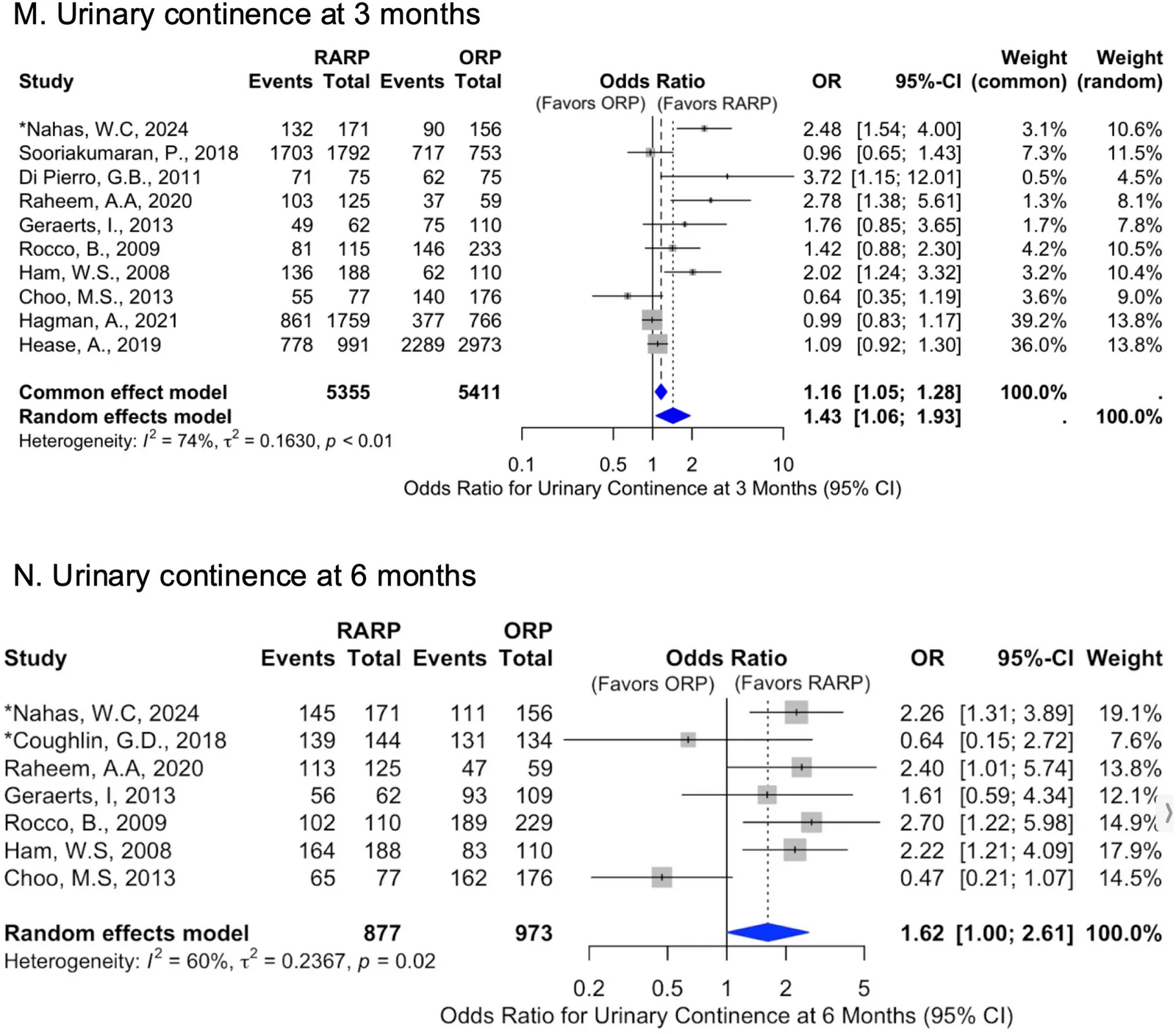

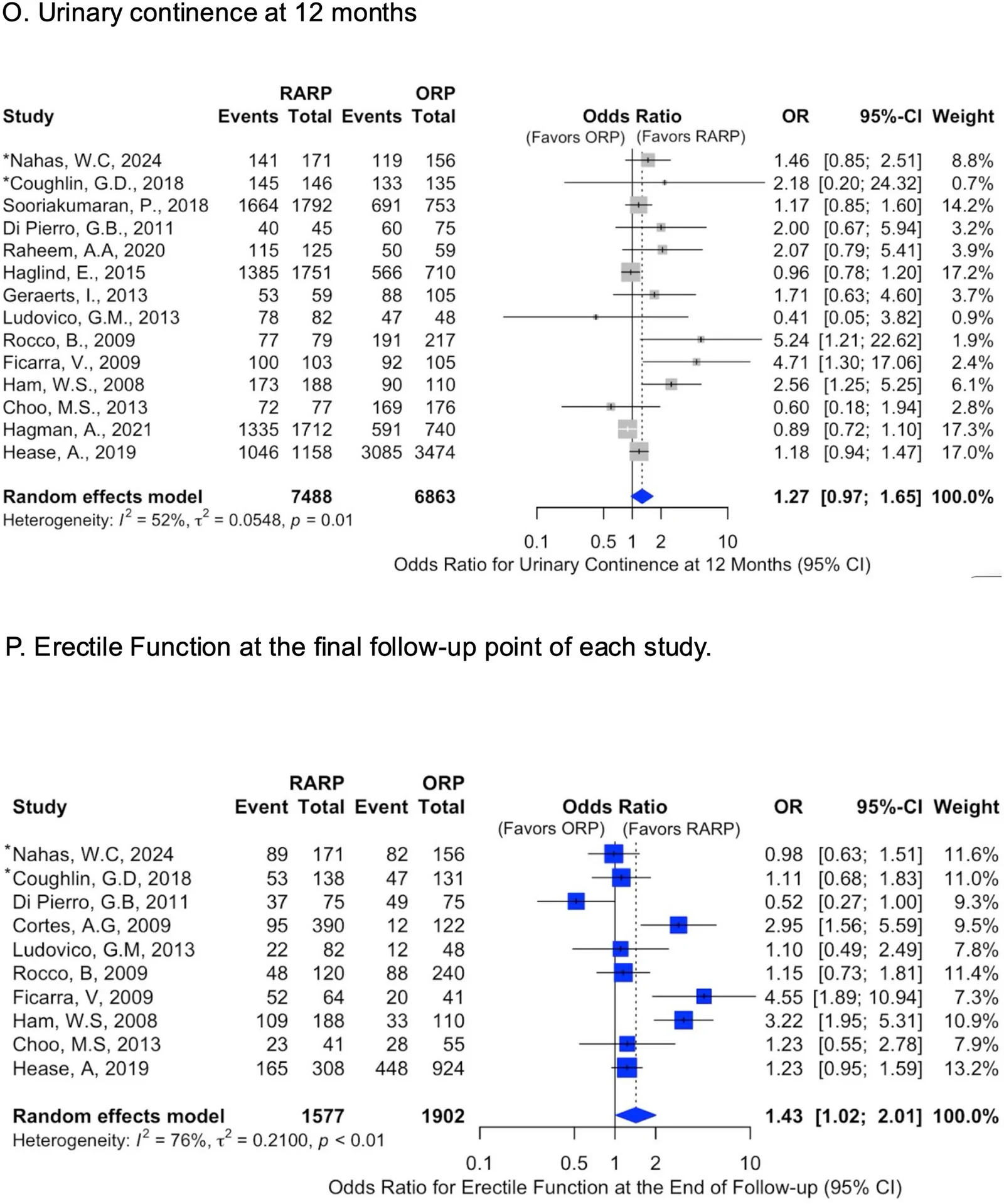

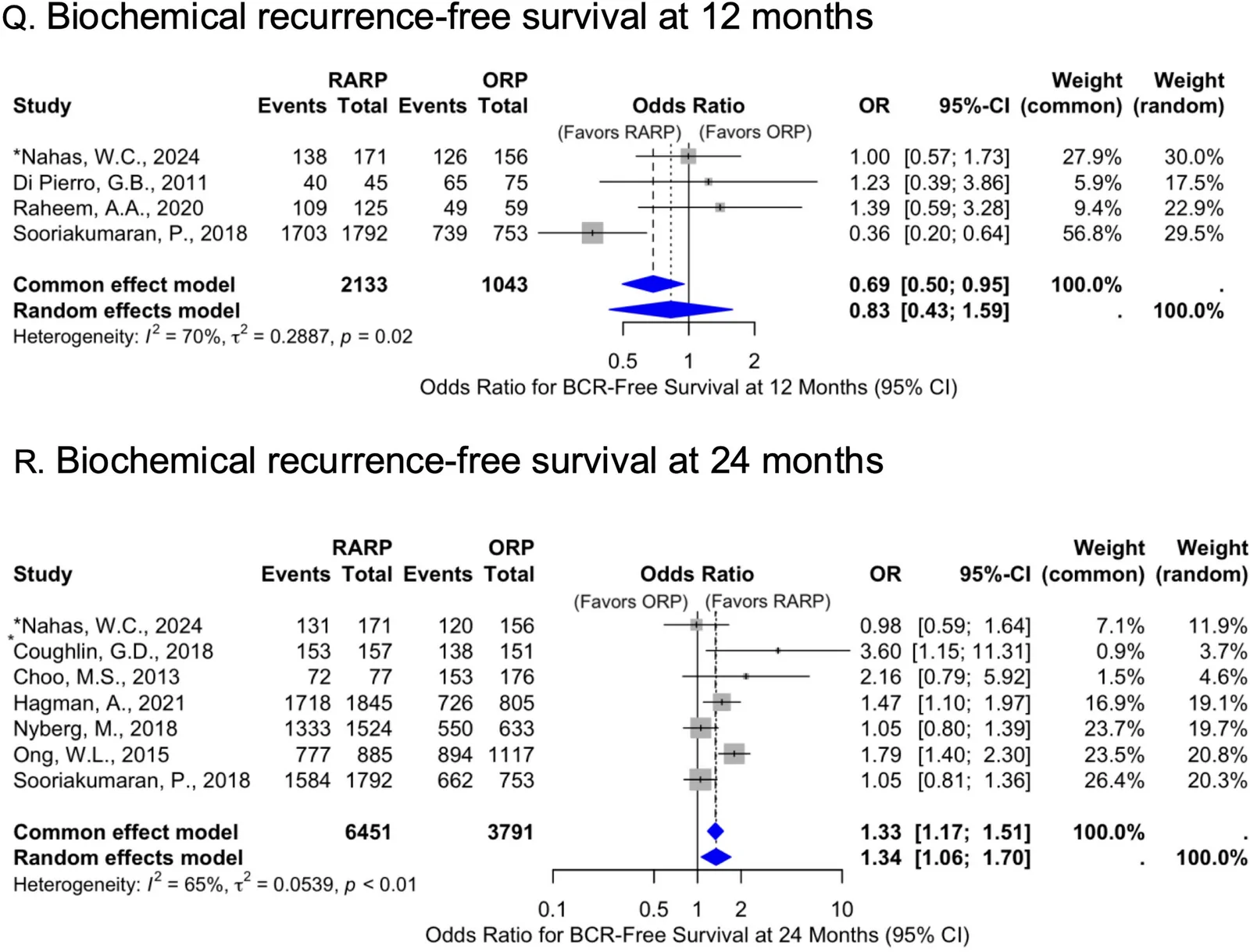
**A** Surgical duration; **B** Estimated blood loss; **C** Intraoperative transfusion rate; **D** Length of hospital stay;** E** Positive surgical margin; **F** Positive surgical margin in pT ≤ 2; **G** Positive surgical margin in ≥ pT3; **H** Nerve-sparing rates; **I** Total postoperative complications; **J** Major postoperative complications; **K** Minor postoperative complications; **L** Urinary continence at 1 month; **M** Urinary continence at 3 months; **N** Urinary continence at 6 months; **O** Urinary continence at 12 months; **P** Erectile function at the final follow-up point of each study; **Q** Biochemical recurrence-free survival at 12 months; **R** Biochemical recurrence-free survival at 24 months

Surgical duration was reported in ten studies [9, 19, 21, 28, 29, 31–35] showing a significant difference between groups and a higher time in the RARP group (MD: 56.61 min, 95%CI 30.11–77.11) with a high heterogeneity (I^2^ = 100%, *p* < 0.0001). (Fig. 3A).

#### Estimated blood loss

Regarding estimated blood loss, ten studies reported these data comparing the groups, and patients undergoing RARP had significantly lower estimated blood loss (MD: − 576 mL, 95%CI − 697.71 to − 455.85), with high heterogeneity among the studies (I^2^ = 96%, *p* < 0.01) [9, 19, 23, 28, 31, 33–37] (Fig. 3B).

#### Intraoperative transfusion rate

As a consequence of the lower estimated blood loss, a significant reduction in transfusion rates was observed in the RARP group (OR: 0.27, 95%CI 0.12–0.65), with high heterogeneity (I^2^ = 95%, *p* < 0.0001) [8, 9, 19, 28, 29, 32–36, 38] (Fig. 3C).

#### Length of hospital stay

The length of hospital stay was described in ten studies [8, 19, 23, 29, 31–33, 35–37]. The meta-analysis demonstrated a significant difference between groups (MD: − 1.44 days, 95%CI − 2.15 to − 0.73), presenting a high heterogeneity (I^2^ = 100%, *p* < 0.0001) (Fig. 3D).

#### Positive surgical margin

Positive surgical margin (PSM) was reported in 20 articles [8, 9, 19, 21–23, 26, 27, 29, 31–37, 39–42]. There was no difference in PSM between the RARP group and the ORP group (OR: 0.91, 95%CI 0.85–0.97) with high heterogeneity (I^2^ = 89%, *p* < 0.01) (Fig. 3E).

The analysis of PSM according to pathological tumor staging did not show statistical significance for the subgroup with pT ≤ 2 (OR: 0.73, 95%CI 0.47–1.16) or for pT3 and above (OR: 1.12, 95%CI 0.86–1.47) (Fig. 3F and 3G).

#### Nerve sparing

Nerve-sparing rates were detailed in sixteen articles [8, 19, 21, 23, 24, 27, 30, 32, 34–37, 39, 40, 42, 43]. For the meta-analysis, nerve-sparing was considered positive if any degree of preservation was achieved. There was an increase in nerve preservation in the RARP group, with statistical significance (OR: 1.95, 95%CI 1.49–2.55), but with high heterogeneity between articles (I^2^ = 83.00%, *p* < 0.01) (Fig. 3H).

### Postoperative complications

Postoperative complications reported as absolute number were discussed in five articles [8, 9, 19, 37, 41] and the classification of complications according to Clavien–Dindo, major and minor was performed in ten [8, 9, 19, 21, 23, 29, 32, 37, 38, 41] and nine [8, 9, 19, 21, 23, 32, 37, 38, 41] studies, respectively. The comparison between groups revealed a reduced risk of absolute complications in the RARP group (OR: 0.59, 95%CI 0.39–0.88) (Fig. 3I), but no statistically significant difference in major complications (OR: 0.69, 95%CI 0.25–1.92) (Fig. 3J) or minor complications (OR: 0.79, 95%CI 0.41–1.54) (Fig. 3K). Analyzing specific complications for each group, the meta-analysis demonstrated that the RARP group was superior in preventing bladder neck stenosis (OR: 0.249, 95%CI 0.103–0.606, *p* = 0.002), but this outcome was reported in just two articles [8, 35].

### Functional outcomes

#### Urinary continence

Postoperative urinary continence, defined as the use of 0—1 safety pads per day, was reported in fourteen studies [8, 9, 20, 21, 23, 27, 31, 33–35, 37, 39, 41, 42] At the one-month follow-up, there was no statistically significant difference between the RARP and ORP groups in terms of continence recovery (OR: 1.57, 95%CI: 0.69–3.59) (Fig. 3L). However, significant improvements in urinary continence were observed at 3 months (OR: 1.43, 95%CI: 1.06–1.93) (Fig. 3M), but not in six months (OR: 1.62, 95%CI: 1.00–1.61) (Fig. 3N) or 12 months follow-up (OR: 1.27, 95%CI: 0.97–1.65) (Fig. 3O) in RARP group.

#### Erectile dysfunction

Erectile dysfunction was reported in ten studies [8, 9, 20, 31, 32, 34, 35, 37, 41], demonstrating a statistically significant higher rate of erectile function recovery in the RARP group compared to the ORP group (OR: 1.43, 95%CI 1.02–2.01) (Fig. 3P). However, the analysis revealed substantial heterogeneity among the included studies (I^2^ = 75.65%, *p* < 0.01), indicating variability in the study designs or populations. For the meta-analysis, the erectile function rates at the final follow-up of each study were used to standardize comparisons across varying time frames, with a median follow-up of 26 months across the studies.

### Oncological outcomes

#### Biochemical recurrence-free survival

Biochemical recurrence-free survival (BCR-free survival) was evaluated after 12 and 24 months of follow-up and was reported in nine studies [9, 20, 21, 23, 26, 34, 39, 42, 43]. After 12 months, there was no significant difference between the groups (OR 0.79, 95%CI 0.32–1.98). However, the RARP group showed a higher BCR-free survival rate after 24 months (OR 1.34, 95%CI 1.06–1.70). Fig. 3Q and 3R present the results.

## Discussion

The recent publication of a high-quality randomized controlled trial [9] has brought new, valuable evidence to the ongoing debate regarding the optimal surgical approach for radical prostatectomy. In this systematic review with meta-analysis, which includes 38,530 patients from all available prospective comparative studies, RARP was associated with longer surgical duration, shorter length of stay, reduced blood loss, lower transfusion rates, a reduction in the bladder neck stenosis rate, faster urinary continence recovery at 3 months and less absolute complications. Additionally, RARP demonstrated higher rates of nerve-sparing, reflecting a higher rate of erectile function recovery, contributing to better functional outcomes and a higher BCR-free survival rate after 24 months. This comprehensive review supports RARP rather than ORP as a safe and effective surgical approach to localized prostate cancer.

We compared our findings with relevant meta-analyses published in 2018, 2019, and 2023 [10, 44–46]. Wang et al. [10] conducted a study similar to ours which enhanced the overall quality of evidence. However, the study was limited by the small number of RCT trials included, which constrained the strength of its conclusions. As a result, we performed an up-to-date systematic review and meta-analysis, including 27 prospective comparative studies with a larger patient cohort, to deliver more current and comprehensive evidence.

Regarding perioperative outcomes, our analysis confirmed that RARP is associated with reduced estimated blood loss, a finding consistent with prior meta-analyses [10, 44, 45]. Furthermore, there is a statistically significant difference in overall complication rates between RARP and ORP, with a lower incidence of bladder neck stenosis in the RARP group. These benefits are largely attributed to the minimally invasive nature of RARP, as previously noted by Nahas et al. [9]. The enhanced visual field and three-dimensional imaging afforded by the robotic console enable greater surgical precision, thus improving hemostasis, particularly in the deep pelvic region where access is challenging due to small anatomical structures [46]. Moreover, the use of pneumoperitoneum plays a crucial role in reducing venous bleeding, a common source of blood loss during radical prostatectomy.

Our analysis further indicated that RARP is linked to superior nerve-sparing outcomes, likely due to the improved visual magnification and precise dissection capabilities of the robotic system. These features enhance procedural observation, which is fundamental in teaching robot-assisted surgeries, enriching trainees’ learning through precise visual detail [47]. Additionally, the increased adoption of robotic systems and growing surgical expertise and the added steps of docking and trocar placement may explain why our study found that RARP had a longer operative time than OR. Additionally, the learning curve of robotic surgery may play a crucial role in explaining this difference too. This contrasts with the findings of Wang et al., who reported no statistically significant difference in operative times between RARP and ORP [10].

Concerning functional outcomes, post-op urinary continence and erectile function are related to many factors involving body mass index, age, and comorbidities [48]. It is also known that an adequate reconstruction of pelvic anatomy, preservation of Retzius space, and nerve-sparing may play a role in achieving this outcome [49, 50].

Our study found statistically significant differences when analyzing urinary continence in 3 months, with RARP showing an improved rate of urinary continence recovery (OR: 1.43, 95%CI 1.06–1.93). When analyzing erectile function recovery, we found a statistically significant benefit to the RARP approach. These findings are consistent with those of Nahas et al. and Wang et al. [9, 10] RARP offers enhanced three-dimensional imaging, more versatile surgical instruments, and greater precision in suturing, all of which may play an important role in promoting better continence and potency outcomes.

The oncological principles remain the cornerstone of radical prostatectomy. Consistent with most prospective studies and systematic reviews, biochemical recurrence in this study was defined as a PSA level of ≥ 0.2 ng/mL. Our analysis demonstrated that RARP was associated with a statistically significant reduction in BCR at 24 months of follow-up, while no significant differences were observed during the short-term period (< 12 months). Considering this data in light of the higher percentage of ISUP grade 4 patients in the ORP group (8.91%) compared to the RARP group (6.82%) is important. However, these results align with previous meta-analyses, reinforcing the oncological safety and efficacy of the robotic approach in prostate cancer management [10, 46, 51].

While our study presents findings from the most recent prospective comparative studies with larger sample sizes addressing perioperative, functional, and oncological outcomes of RARP and ORP, some limitations must be acknowledged. Our meta-analysis included only two randomized controlled trials, and the included studies had considerable methodological variability, particularly in the study design, data collection, and reporting standards. Several additional limitations of this review should be acknowledged. First, the included studies exhibited considerable heterogeneity, likely stemming from variations in surgical techniques, surgeon expertise, patient demographics, and sample sizes. Second, the lack of reported data precluded robust statistical analyses of long-term functional outcomes. Finally, we were unable to assess potential confounders associated with reduced bladder neck stricture rates—such as catheterization duration or the implementation of fascia-sparing techniques—due to insufficient reporting in the primary studies.

Another limitation of this study is the absence of PSMA-based imaging for predicting and localizing histologic malignancy within the prostate. Preoperative assessment could potentially be improved by incorporating Ga-PSMA PET alongside mpMRI. PSMA PET-CT offers higher sensitivity and specificity for detecting metastatic disease, which may directly influence surgical decision-making, the indication for extended pelvic lymph node dissection, and patient selection for multimodal therapy.

Lastly, more comprehensive long-term follow-up data are needed, especially concerning tumor prognosis and cancer-specific mortality, as shorter follow-up periods may limit the confidence in these outcomes.

## Conclusion

In conclusion, this systematic review and meta-analysis demonstrates that RARP has advantages over ORP. RARP is associated with superior perioperative outcomes (including reduced blood loss, lower transfusion rates, and shorter hospital stays), lower overall complication rates, and higher nerve-sparing rates. Moreover, it yields better functional recovery of erectile function and continence, alongside improved long-term BCR rates. It is a safe and effective surgical technique for treating clinically localized PCa.

### Technical blurb

A systematic review and meta-analysis shows that RARP shows reduced blood loss, fewer complications, faster recovery, and improved functional outcomes compared with ORP, while maintaining comparable oncologic control, based on a review of 27 studies including 38,530 patients.

## Supplementary Information

Below is the link to the electronic supplementary material.

Supplementary file1 (DOCX 669 KB)

## Data Availability

No datasets were generated or analyzed during the current study.
