# Supplementary material for: Comparative functional, perioperative and oncological outcomes of robot-assisted and open radical prostatectomy: a systematic review and meta-analysis
Source: Int Urol Nephrol. 2026 Jun 2;58(11):4575–93. doi: 10.1007/s11255-026-05182-5 (PMC13694353; doi:10.1007/s11255-026-05182-5)
Supplement: Supplementary file 1 [file 11255_2026_5182_MOESM1_ESM.docx]

**SUPPLEMENTARY MATERIAL 1 : SEARCH STRATEGY**

(("Prostatectomies" OR "Prostatectomy, Suprapubic" OR "Prostatectomies, Suprapubic" OR "Suprapubic Prostatectomy" OR "Prostatectomy, Retropubic" OR "Prostatectomies, Retropubic" OR "Retropubic Prostatectomies" OR "Retropubic Prostatectomy") AND ("Procedure, Robotic Surgical" OR "Robotic Surgical Procedure" OR "Surgical Procedure, Robotic" OR "Robot Surgery" OR "Robot Surgeries" OR "Surgery, Robot" OR "Robot-Assisted Surgery" OR "Robot Assisted Surgery" OR "Robot-Assisted Surgeries" OR "Surgery, Robot-Assisted" OR "Robot-Enhanced Procedures" OR "Procedure, Robot-Enhanced" OR "Robot Enhanced Procedures" OR "Robot-Enhanced Procedure" OR "Surgical Procedures, Robotic" OR "Robotic-Assisted Surgery" OR "Robotic Assisted Surgery" OR "Robotic-Assisted Surgeries" OR "Surgery, Robotic-Assisted" OR "Robot-Enhanced Surgery" OR "Robot Enhanced Surgery" OR "Robot-Enhanced Surgeries" OR "Surgery, Robot-Enhanced" OR "RARP"))

**SUPPLEMENTARY MATERIAL 2 - FUNNEL PLOT / EGGER TEST**

**SUPPLEMENTARY MATERIAL 3 – GRADE CLASSIFICATION**

**Author(s): Caio Vinicius Suartz, Mateus Silva Santos Costa, Caio Felipe Araujo Matalani, Marcelo Ribeiro da Rocha, Henrique Laurent, Lepine, Roberto Iglesias Lopes, Thalita Bento Talizin, Pedro Luis Furlam, José Bessa Júnior, Walid Shahrour, Walid Shabana, Axel S Merseburger, Leonardo Oliveira Reis, Maurício Dener Cordeiro, William Carlos Nahas, Paul Toren, Leopoldo Alves Ribeiro-Filho , Guillaume Ploussard,Vicent Fradet Frédéric Pouliot**

**Question:** RARP compared to ORP for localized prostate cancer treatment

**Setting:**

**Bibliography:**

| **Certainty assessment** | | | | | | | **№ of patients** | | **Effect** | | **Certainty** | **Importance** |
| --- | --- | --- | --- | --- | --- | --- | --- | --- | --- | --- | --- | --- |
| **№ of studies** | **Study design** | **Risk of bias** | **Inconsistency** | **Indirectness** | **Imprecision** | **Other considerations** | **RARP** | **ORP** | **Relative (95% CI)** | **Absolute (95% CI)** |  |  |
| **Surgical time (follow-up: range 3 months to 4 years)** | | | | | | | | | | | | |
| 10 | non-randomised studies | not serious | very serious^a^ | not serious | very serious^b^ | none | 7148 | 8942 | - | SMD **1.496 SD higher** (0.9 higher to 2.092 higher) | ⨁◯◯◯ Very low^a,b^ | IMPORTANT |
| **Blood Loss (follow-up: range 3 months to 4 years; assessed with: ml; Scale from: 117.5 to 1410)** | | | | | | | | | | | | |
| 10 | non-randomised studies | not serious | very serious^a^ | not serious | not serious | publication bias strongly suspected strong association^c^ | 7043 | 9247 | - | SMD **2.377 SD lower** (2.815 lower to 1.94 lower) | ⨁⨁◯◯ Low^a,c^ | IMPORTANT |
| **Transusion Rate (follow-up: range 3 months to 4 years)** | | | | | | | | | | | | |
| 10 | non-randomised studies | not serious | very serious^d^ | not serious | not serious | publication bias strongly suspected strong association^c^ | 183/6700 (2.7%) | 522/11263 (4.6%) | **OR 0.248** (0.089 to 0.687) | **34 fewer per 1000** (from 42 fewer to 14 fewer) | ⨁⨁◯◯ Low^c,d^ | IMPORTANT |
| **Hospital Stay (follow-up: range 3 months to 4 years; assessed with: Days; Scale from: 1.55 to 8.1)** | | | | | | | | | | | | |
| 10 | non-randomised studies | not serious | very serious^a^ | not serious | not serious | none | 7764 | 9329 | - | SMD **1.289 SD lower** (2.015 lower to 0.564 lower) | ⨁⨁◯◯ Low^a^ | NOT IMPORTANT |
| **Positive Surgical Margin (follow-up: range 3 months to 4 years)** | | | | | | | | | | | | |
| 20 | non-randomised studies | not serious | very serious^a^ | not serious | serious^e^ | publication bias strongly suspected^c^ | 3254/16855 (19.3%) | 2527/13816 (18.3%) | **OR 0.871** (0.710 to 1.060) | **20 fewer per 1000** (from 46 fewer to 9 more) | ⨁◯◯◯ Very low^a,c,e^ | CRITICAL |
| **Positive Surgical Margin p ≤ T2 (follow-up: range 3 months to 4 years)** | | | | | | | | | | | | |
| 11 | non-randomised studies | not serious | very serious^a^ | not serious | serious^e^ | none | 602/7202 (8.4%) | 731/8243 (8.9%) | **OR 0.734** (0.465 to 1.158) | **22 fewer per 1000** (from 45 fewer to 13 more) | ⨁◯◯◯ Very low^a,e^ | IMPORTANT |
| **Positive Surgical Margin p ≥ T3 (follow-up: range 3 months to 4 years)** | | | | | | | | | | | | |
| 11 | non-randomised studies | not serious | serious^f^ | not serious | very serious^b^ | none | 795/5373 (14.8%) | 898/5088 (17.6%) | **OR 1.101** (0.879 to 1.379) | **14 more per 1000** (from 18 fewer to 52 more) | ⨁◯◯◯ Very low^b,f^ | IMPORTANT |
| **Nerve-Sparing (follow-up: range 3 months to 4 years)** | | | | | | | | | | | | |
| 16 | non-randomised studies | not serious | very serious^a^ | not serious | not serious | publication bias strongly suspected^c^ | 12100/14120 (85.7%) | 10262/12135 (84.6%) | **OR 1.930** (1.554 to 2.397) | **68 more per 1000** (from 49 more to 84 more) | ⨁◯◯◯ Very low^a,c^ | IMPORTANT |
| **Complication (follow-up: range 3 months to 4 years)** | | | | | | | | | | | | |
| 5 | non-randomised studies | very serious^g^ | not serious | not serious^e^ | not serious | publication bias strongly suspected^c^ | 47/701 (6.7%) | 65/570 (11.4%) | **OR 0.587** (0.392 to 0.878) | **44 fewer per 1000** (from 66 fewer to 12 fewer) | ⨁◯◯◯ Very low^c,e,g^ | CRITICAL |
| **Major Complication (follow-up: range 3 months to 4 years)** | | | | | | | | | | | | |
| 10 | non-randomised studies | not serious | very serious^a^ | not serious | serious^e^ | publication bias strongly suspected^c^ | 113/2735 (4.1%) | 90/3802 (2.4%) | **OR 0.678** (0.194 to 2.376) | **7 fewer per 1000** (from 19 fewer to 31 more) | ⨁◯◯◯ Very low^a,c,e^ | CRITICAL |
| **Minor Complication (follow-up: range 3 months to 4 years)** | | | | | | | | | | | | |
| 9 | non-randomised studies | not serious | very serious^a^ | not serious | serious^e^ | publication bias strongly suspected^c^ | 350/2223 (15.7%) | 268/3590 (7.5%) | **OR 0.785** (0.297 to 2.077) | **15 fewer per 1000** (from 51 fewer to 69 more) | ⨁◯◯◯ Very low^a,c,e^ | IMPORTANT |
| **Urinary Continence at 1 Month (follow-up: mean 1 months)** | | | | | | | | | | | | |
| 6 | non-randomised studies | not serious | very serious^a^ | not serious | serious^e^ | none | 632/1999 (31.6%) | 1365/4895 (27.9%) | **OR 1.572** (0.848 to 2.915) | **99 more per 1000** (from 32 fewer to 251 more) | ⨁◯◯◯ Very low^a,e^ | IMPORTANT |
| **Urinary Continence at 3 Month (follow-up: mean 3 months)** | | | | | | | | | | | | |
| 10 | non-randomised studies | not serious | very serious^a^ | not serious | not serious | publication bias strongly suspected^c^ | 5768/6451 (89.4%) | 3243/3791 (85.5%) | **OR 1.396** (1.087 to 1.793) | **37 more per 1000** (from 10 more to 58 more) | ⨁◯◯◯ Very low^a,c^ | CRITICAL |
| **Urinary Continence at 6 Month (follow-up: mean 6 months)** | | | | | | | | | | | | |
| 7 | non-randomised studies | serious^h^ | very serious^a^ | not serious | not serious | none | 784/877 (89.4%) | 816/973 (83.9%) | **OR 1.618** (1.002 to 2.614) | **55 more per 1000** (from 0 fewer to 93 more) | ⨁◯◯◯ Very low^a,h^ | CRITICAL |
| **Urinary Continence at 12 Month (follow-up: mean 12 months)** | | | | | | | | | | | | |
| 14 | non-randomised studies | not serious | very serious^a^ | not serious | not serious | none | 6424/7488 (85.8%) | 5972/6863 (87.0%) | **OR 1.267** (1.027 to 1.563) | **24 more per 1000** (from 3 more to 43 more) | ⨁⨁◯◯ Low^a^ | CRITICAL |
| **Erectile Function (follow-up: range 3 months to 4 years)** | | | | | | | | | | | | |
| 10 | non-randomised studies | not serious | very serious^a^ | not serious | not serious | none | 693/1577 (43.9%) | 819/1902 (43.1%) | **OR 1.428** (1.017 to 2.006) | **89 more per 1000** (from 4 more to 172 more) | ⨁⨁◯◯ Low^a^ | CRITICAL |
| **Biochemical Recurrence-free Survival at 12 months (follow-up: mean 12 months)** | | | | | | | | | | | | |
| 4 | non-randomised studies | serious^i^ | very serious^a^ | not serious | very serious^b^ | none | 1990/2133 (93.3%) | 979/1043 (93.9%) | **OR 0.83** (0.42 to 1.63) | **12 fewer per 1000** (from 73 fewer to 23 more) | ⨁◯◯◯ Very low^a,b,i^ | CRITICAL |
| **Biochemical Recurrence-free Survival at 24 months (follow-up: mean 24 months)** | | | | | | | | | | | | |
| 7 | non-randomised studies | not serious | very serious^a^ | not serious | not serious | none | 5768/6451 (89.4%) | 3243/3791 (85.5%) | **OR 1.340** (1.054 to 1.720) | **33 more per 1000** (from 6 more to 55 more) | ⨁⨁◯◯ Low^a^ | CRITICAL |

**CI:** confidence interval; **OR:** odds ratio; **SMD:** standardised mean difference

#### Explanations

a. There is substantial variation in the point estimates of the studies and the confidence intervals, with an I² greater than 50% and statistical significance. Moreover, no subgroup analysis or meta-regression was performed to explain this effect.

b. The point estimate observed in the control group was higher, and the confidence interval includes the null value.

c. Egger's Test p < 0.05

d. There is slight variation in the point estimates of the studies and the confidence intervals, in addition to presenting an I² = 94.25% and P < 0.0001. Moreover, no subgroup analysis or meta-regression was performed to explain this effect.

e. The point estimate of the outcome observed in the intervention group was better, but the confidence interval includes the null value.

f. There is slight variation in the point estimates of the studies and the confidence intervals, in addition to presenting an I² = 64.64% and P = 0.0025. Moreover, no subgroup analysis or meta-regression was performed to explain this effect.

g. The weight of the studies classified as high risk of bias, according to the RoB-2 tool for RCTs and the ROBINS-I tool for prospective cohorts, was 36.37

h. The weight of the studies classified as high risk of bias, according to the RoB-2 tool for RCTs and the ROBINS-I tool for prospective cohorts, was 26.74

i. The weight of the studies classified as high risk of bias, according to the RoB-2 tool for RCTs and the ROBINS-I tool for prospective cohorts, was 29.68
